# A 3-*O*-sulfated heparan sulfate dodecasaccharide (12-mer) suppresses thromboinflammation and attenuates early organ injury following trauma and hemorrhagic shock

**DOI:** 10.3389/fimmu.2023.1158457

**Published:** 2023-04-14

**Authors:** Maria del Pilar Huby Vidaurre, Baron K. Osborn, Kaylie D. Lowak, Michelle M. McDonald, Yao-Wei W. Wang, Veda Pa, Jillian R. Richter, Yongmei Xu, Katelyn Arnold, Jian Liu, Jessica C. Cardenas

**Affiliations:** ^1^ Center for Translational Injury Research, Department of Surgery, McGovern Medical School at The University of Texas Health Science Center at Houston, Houston, TX, United States; ^2^ Department of Pathology and Laboratory Medicine, McGovern Medical School at The University of Texas Health Science Center at Houston, Houston, TX, United States; ^3^ Department of Surgery, Division of Trauma and Acute Care Surgery, The University of Alabama at Birmingham, Birmingham, AL, United States; ^4^ Eshelman School of Pharmacy, Division of Chemical Biology and Medicinal Chemistry, The University of North Carolina at Chapel Hill, Chapel Hill, NC, United States

**Keywords:** thromboinflammation, heparan sulfate, traumatic injury, hemorrhagic shock, organ injury

## Abstract

**Introduction:**

Dysregulated inflammation and coagulation are underlying mechanisms driving organ injury after trauma and hemorrhagic shock. Heparan sulfates, cell surface glycosaminoglycans abundantly expressed on the endothelial surface, regulate a variety of cellular processes. Endothelial heparan sulfate containing a rare 3-*O*-sulfate modification on a glucosamine residue is anticoagulant and anti-inflammatory through high-affinity antithrombin binding and sequestering of circulating damage-associated molecular pattern molecules. Our goal was to evaluate therapeutic potential of a synthetic 3-*O*-sulfated heparan sulfate dodecasaccharide (12-mer, or dekaparin) to attenuate thromboinflammation and prevent organ injury.

**Methods:**

Male Sprague-Dawley rats were pre-treated subcutaneously with vehicle (saline) or dekaparin (2 mg/kg) and subjected to a trauma/hemorrhagic shock model through laparotomy, gut distention, and fixed-pressure hemorrhage. Vehicle and dekaparin-treated rats were resuscitated with Lactated Ringer’s solution (LR) and compared to vehicle-treated fresh-frozen-plasma-(FFP)-resuscitated rats. Serial blood samples were collected at baseline, after induction of shock, and 3 hours after fluid resuscitation to measure hemodynamic and metabolic shock indicators, inflammatory mediators, and thrombin-antithrombin complex formation. Lungs and kidneys were processed for organ injury scoring and immunohistochemical analysis to quantify presence of neutrophils.

**Results:**

Induction of trauma and hemorrhagic shock resulted in significant increases in thrombin-antithrombin complex, inflammatory markers, and lung and kidney injury scores. Compared to vehicle, dekaparin treatment did not affect induction, severity, or recovery of shock as indicated by hemodynamics, metabolic indicators of shock (lactate and base excess), or metrics of bleeding, including overall blood loss, resuscitation volume, or hematocrit. While LR-vehicle-resuscitated rodents exhibited increased lung and kidney injury, administration of dekaparin significantly reduced organ injury scores and was similar to organ protection conferred by FFP resuscitation. This was associated with a significant reduction in neutrophil infiltration in lungs and kidneys and reduced lung fibrin deposition among dekaparin-treated rats compared to vehicle. No differences in organ injury, neutrophil infiltrates, or fibrin staining between dekaparin and FFP groups were observed. Finally, dekaparin treatment attenuated induction of thrombin-antithrombin complex and inflammatory mediators in plasma following trauma and hemorrhagic shock.

**Conclusion:**

Anti-thromboinflammatory properties of a synthetic 3-*O*-sulfated heparan sulfate 12-mer, dekaparin, could provide therapeutic benefit for mitigating organ injury following major trauma and hemorrhagic shock.

## Introduction

Organ injury and subsequent development of multiple organ dysfunction syndrome (MODS) is a leading cause of morbidity and mortality following major trauma and hemorrhagic shock (T/HS) (1). While the underlying pathogenesis of MODS after traumatic hemorrhage remains poorly understood, pronounced activation of the coagulation and inflammation systems is evident early in the clinical course and is a known mediator of organ injury in the critically ill (2–4). The vascular endothelium is a key regulator of thromboinflammation through its expression of an extensive network of anticoagulant and anti-adhesive molecules (5). These include heparan sulfate proteoglycans (HSPGs), which regulate leukocyte-endothelial interactions, barrier function, and the overall anticoagulant tone of the endothelium (6, 7). The biological function of HSPGs is determined, in large part, by sulfation patterns on the heparan sulfate polysaccharides (6, 7). In particular, heparan sulfate with the specific pentasaccharide sequence containing a central 3-*O*-sulfated glucosamide enables high-affinity binding to circulating antithrombin-III (AT), leading to inhibition of coagulation factor Xa and thrombin (8–10). This interaction between AT and 3-*O*-sulfated heparan sulfate containing HSPGs (3-OS-HSPG) both disrupts coagulation at the endothelial surface and elicits anti-inflammatory effects through induction of prostacyclin synthesis and inhibition of NFκB activation (9, 11, 12). In addition, recent data demonstrate that 3-OS-HSPG in circulation can accelerate AT’s anticoagulant activity and also directly bind and sequester circulating damage-associated molecular pattern molecules, including high mobility group box-1 (HMGB-1) (13). For these reasons, 3-OS-HSPG is an important anti-thromboinflammatory molecule and has reported therapeutic benefits in a rodent model of ischemia-induced liver injury (7, 13).

T/HS-induced shedding of HSPGs is strongly associated with organ injury, MODS, and death (14–16); however, the potential therapeutic effects of HSPG molecules such as 3-OS-HSPG have not been examined in the context of T/HS-associated organ injury. The objective of this study was to determine if a synthetic 3-*O*-sulfated heparan sulfate 12-mer, dekaparin, could prevent thromboinflammation and organ injury in a murine model of T/HS. We hypothesized that dekaparin treatment would reduce systemic markers of coagulation, inflammation, and attenuate multiple organ injury after induction of T/HS.

## Materials and methods

### Chemoenzymatic synthesis of dekaparin

**Figure 1:**
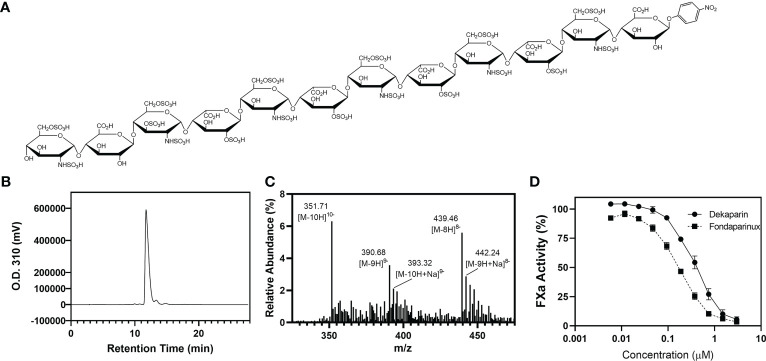
Dekaparin chemical structure, purity, and anti-FXa activity. **(A)** Dekaparin chemical structure prepared by chemoenzymatic synthesis. **(B)** HPLC chromatogram of dekaparin. **(C)** Electrospray ionization mass spectrometry (ESI-MS) spectrum collected in negative mode. Molecular weight (MW) determined from ESI-MS analysis is 3525.35 +/- 1.6 Da, which is close to the calculated MW of 3523.76 Da. **(D)** Anti-FXa activity illustrates dekaparin’s anticoagulant activity. Fondaparinux is included as a control. HPLC, high performance liquid chromatography; FXa, factor Xa.

The chemical structure of dekaparin is provided in **Figure 1A**. The synthesis of dekaparin was completed using the chemoenzymatic approach described previously (17). Briefly, the synthesis was initiated from a monosaccharide *p*-nitrophenyl glucuronide. The monosaccharide was elongated to the desired size of oligosaccharides using heparosan synthase 2. The oligosaccharides were then modified by a series of heparan sulfate biosynthetic enzymes, including *N*-sulfotransferase, C_5_-epimerase (C_5_-epi), 2-*O*-sulfotransferase (2-OST), 6-*O*-sulfotransferase, and 3-*O*-sulfotransferase 1. All biosynthetic enzymes were expressed and purified in-house. All enzymes, with the exception of C_5_-epi and 2-OST, were expressed in *Escherichia coli* and purified by appropriate affinity chromatography as described previously (18, 19). Recombinant C_5_-epi and 2-OST were expressed in insect cells using the Bac-to-Bac baculovirus expression approach (Invitrogen) to obtain high expression levels (17). Co-factors and monosaccharide building blocks, including 3’-phosphoadenosine 5’-phosphosulfate (PAPS), uridine 5’-diphosphoglucuronic acid (UDP-GlcA), and uridine 5’-diphospho N-trifluoroacetyl glucosamine (UDP-GlcNTFA), were all synthesized in-house using enzymatic approaches as described previously (17).

At every synthesis step, the products were monitored by Shimadzu high-performance liquid chromatography (HPLC) equipped with a Propac column (Propac PA1, 10 μm, 9 mm × 250 mm, Thermo Fisher Scientific). Buffer A was 20 mM NaAcO (pH 5.0), and buffer B was 2 M NaCl with 20 mM NaAcO (pH 5.0). A linear gradient was used to separate the starting material and product. Different gradients were chosen by the sulfation degree of the compound. The ultraviolet light detector was set at 310 nm to monitor the oligosaccharide. Chemical purity was confirmed by high-resolution anion exchange HPLC (**Figure 1B**). The product was confirmed by electrospray ionization mass spectrometry (ESI-MS). The measured molecular weight (MW) of dekaparin was determined by ESI-MS to be 3522.8 Da, close to the calculated MW of 3523.8 Da (**Figure 1C**). The ESI-MS data were acquired and processed using Xcalibur 1.3 (Thermo Fischer Scientific).

Endotoxin was removed from dekaparin by centrifugation (Amicon Ultra-15, Ultracel-100k; Merck Millipore). The absence of endotoxin in the purified dekaparin was confirmed by the Limulus Amebocyte Lysate kit (sensitivity=0.125 EU/mL; Associates of Cape Cod, Inc.) according to manufacturer’s protocol.

The pharmacokinetics of dekaparin have been previously reported (20). Plasma concentrations of dekaparin peaked approximately 30-45 minutes after subcutaneous injection and decreased after approximately 60 minutes. Pharmacokinetic profiles of dekaparin were found to be similar to those of the low-molecular-weight heparin, enoxaparin.

### Determination of *in vitro* FXa activity

Human Factor Xa (FXa) (Enzyme Research Laboratories) was diluted to 50 U/mL with phosphate-buffered saline (PBS). The chromogenic substrate S-2765 (Diapharma) was diluted to 1 mg/mL in water. Antithrombin-III (Cutter Biologics) was diluted to 0.034 mg/mL in 1 mg/mL bovine serum albumin (BSA)/PBS. Dekaparin and fondaparinux were serially diluted in PBS (0.005 - 3 µM). Ten μL of samples were incubated with 60 μL of antithrombin for 2 min at room temperature. Next, 100 μL of FXa was added and incubated for 3 min at room temperature. Thirty μL of S-2765 substrate (Diapharma) was added, and the absorbance of the reaction mixture was measured at 405 nm every 10 seconds for 2 min. PBS served as a control sample. The maximum slope for each sample was converted to percent FXa activity by dividing by the maximum slope for the control sample (**Figure 1D**).

### Animal subjects

All experimental procedures were approved by the Institutional Animal Care and Use Committee of The University of Texas Health Science Center at Houston (AWC-21-0088) and were performed in accordance with the Animal Research: Reporting of *In Vivo* Experiments (ARRIVE) guidelines (21). Power analyses were conducted on a pilot cohort of N=3 LR-vehicle and N=3 LR-dekaparin-treated animals to determine samples sizes necessary to detect significant differences in lung injury scores, resulting in a sample size of N=9 animals per group. All animals were randomly allocated to treatment groups using Excel. Adult male Sprague-Dawley rats 10 to 12 weeks old, weighing between 225 and 350 g were housed in an accredited animal facility and acclimatized for 3 days prior to experimentation. Rodents were housed in a semi-barrier facility in individually ventilated cages with irradiated bedding and received irradiated food and water. Food and water were available *ad libitum*. For environmental enrichment, rats were housed socially (2 per cage) with cotton nestles and polycarbonate tunnels. Both body weight and health were monitored daily. We utilized a well-described model of T/HS (22–24). Briefly, rats were anesthetized with inhalatory isoflurane, USP (Piramal Enterprises Ltd) (2%) in 100% O_2_. Body temperature was continuously monitored through a rectal thermometer and maintained at 37°C using a heating pad (TCAT-2LV controller Thermocoupler, Physitemp). The right femoral superficial artery and vein were isolated and cannulated using heparin-washed PE-50 polyethylene catheters. The arterial blood pressure (BP), mean arterial pressure (MAP), and heart rate were monitored continuously (PowerLab 4/10, AD Instruments) and digitally recorded throughout the study period (LabChart Pro, AD Instruments). Once the catheterization was complete, rats were left to hemodynamically stabilize for 15 minutes, after which baseline blood samples were collected (1 mL). This was followed by subcutaneous (SQ) injection of either vehicle (normal saline) or dekaparin (2 mg/kg) in a total volume of 1 mL. Experimental treatments were administered prior to induction of T/HS in agreement with past reports examining the protective effects of heparin molecules for preventing organ injury following burn, sepsis, and mechanical ventilation (25–29). Further, our past studies using rat models have demonstrated that thrombin generation is very rapidly induced following major hemorrhage (23). Thus, we sought to prevent upregulation of thrombin production through dekaparin administration prior to injury.

**Figure 2:**
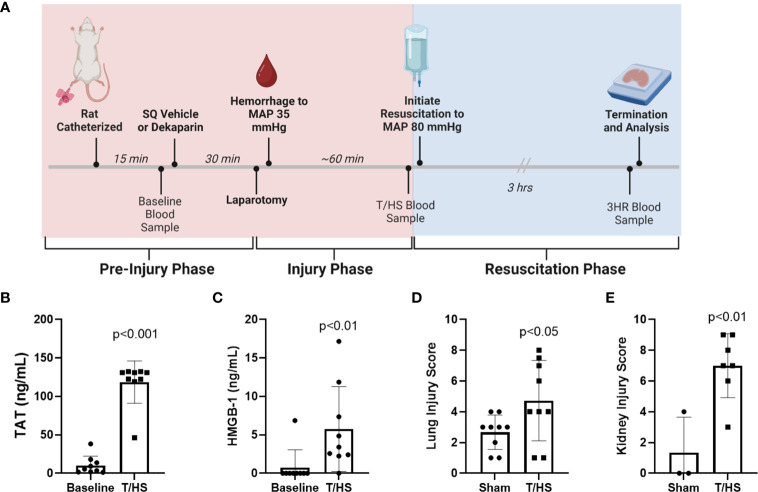
Rat model of trauma and hemorrhagic shock (T/HS) induces systemic markers of thromboinflammation and multiple organ injury. **(A)** Experimental schema including treatment administration, induction of T/HS through laparotomy and controlled hemorrhage, resuscitation, and sampling timeline. **(B)** Thrombin-antithrombin (TAT) complex and **(C)** high mobility group box-1 (HMGB-1) levels were measured after T/HS by enzyme-linked immunosorbent assay (N=9 per group). Data were compared using paired *t* tests. **(D)** Lungs (N=9 per group) and **(E)** kidneys (N=3 sham and N=7 T/HS) were collected 3 hours after resuscitation (3HR) with LR and stained with hematoxylin and eosin and degree of organ injury scored by a blinded pathologist. Data presented as mean with standard deviation. SQ, subcutaneous; MAP, mean arterial pressure; LR, Lactated Ringer's.

Thirty minutes after injection, rats were subjected to 3 cm midline laparotomy and exteriorization and distension of the small intestine to induce tissue trauma. The intestines were repositioned, and the abdominal incision was closed. Animals were then subjected to a model of fixed-pressure hemorrhagic shock through rapid, controlled hemorrhage to a target MAP of 35 mmHg (**Figure 2A**). Rats were maintained at this MAP for approximately 60 minutes through fluid infusion, as needed, until reaching a state of decompensation, defined as loss of the ability to maintain a MAP of 35 mmHg, indicating severe HS and necessitating resuscitation. A second blood sample was collected at this time (1 mL; end of T/HS sample). Rats were resuscitated with either Lactated Ringer’s solution (LR; Braun Medical Inc.) or fresh frozen plasma (FFP; Innovative Research Inc.) to restore a target MAP of 80 mmHg. Rats were maintained and observed under anesthesia for 3 hours, after which a final blood sample was collected (3 hours after resuscitation sample, 3HR), the animal euthanized, and tissues harvested (**Figure 2A**). Lungs were harvested from the entire study cohort. Kidney collection was incorporated after study initiation. Sham rats were subjected to anesthesia, femoral artery and vein catheterization, and repeated blood sampling.

### Metabolic assessment of shock

Total blood volumes were calculated based on an estimated blood volume of 6.5 mL per 100-gram body weight reported for male rats (30). This was used to determine the percent blood volume withdrawn. Base excess (mmol/L), lactate (mmol/L), pH, and hematocrit (%) were assessed using an iSTAT-1 Analyzer (Abbott) at baseline, after induction of T/HS, and at 3HR to further define the presence of shock, effectiveness of resuscitation, and impact of dekaparin on induction or recovery from T/HS.

### Organ histopathology/immunohistochemistry

Paraffin-embedded lung and kidney sections were stained with hematoxylin and eosin (H&E) and analyzed for evidence of organ injury by a blinded, board-certified pathologist under a 40x objective. Organs that experienced issues during harvest or processing were excluded. Lung and kidney injury were defined using previously described (22), 4-point scoring scales (see **Supplemental Tables 1** [lung] and **2** [kidney]) (31–33). The total organ injury score was determined by adding all values assigned for each individual scoring parameter. For lungs, scores ranged from 0 to 18. For kidneys, scores ranged from 0 to 12. To assess organ neutrophil infiltration and intravascular fibrin deposition, we performed immunohistochemistry as previously described (34) using an anti-myeloperoxidase (MPO) antibody (Abcam ab208670; 1:750) or an anti-fibrin antibody (GeneTex GTX19079; 1:500). Immunostaining was quantified by taking 3 random images of each lung and kidney (cortex). The number of MPO-positive cells per field of view was counted, and fibrin staining density was quantified by NIS Elements software (version 4.5) by an individual blinded to the treatment groups.

### Plasma analysis

Plasma was prepared by centrifuging blood at 4,000 x *g* for 15 minutes at room temperature and aliquoted and frozen at -80°C until analysis. Thrombin-antithrombin (TAT) complex levels were measured at the end of the experiment by enzyme-linked immunosorbent assay (ELISA) (Siemens Enzygnost TAT Micro Kit). Plasma inflammatory mediators were measured using the Bio-Plex Pro Rat Cytokine 23-Plex Assay and HMGB-1 ELISA (IBL-International). Plasma creatinine was measured by ELISA (Invitrogen). All samples were performed in duplicate. Sample sizes vary due to differences in plasma sample collection volumes.

### Statistics

Quantile-quantile plots were used to assess normal distribution of all data. Data are presented as means, with bars representing standard deviation. Statistical analyses were performed using GraphPad Prism Version 9.5 (Dotmatics). Statistical significance between sham and T/HS groups was determined by two-tailed, unpaired Student’s *t* test. Statistical significance between baseline and T/HS timepoints from serial samples was determined by two-tailed paired Student’s *t* test. Differences between multiple groups in single time point parameters were determined using repeated measures one-way analysis of variance with Tukey adjustment for multiple comparisons.

## Results

### T/HS induces a pro-thrombinflammatory state and subsequent organ injury

The T/HS model experimental schema depicting timeline and sample collection is provided in **Figure 2A**. A total of 9 animals were randomized to each group. One animal randomized to the FFP group died prior to resuscitation and was therefore excluded from analysis, leaving 8 animals in this group. Hemodynamic changes that confirm hemorrhagic shock include decreased MAP at the end of the hemorrhage period (T/HS) when compared to baseline (baseline 84.1 ± 7.5 mmHg vs. T/HS 28.5 ± 6.7 mmHg; p<0.0001), and reduced hematocrit (baseline 48.9 ± 4.0% vs. T/HS 31.1 ± 2.5%; p<0.0001). The metabolic changes induced by T/HS include acidosis reflected by decreased base excess (BE) (baseline 3.9 ± 1.3 mmol/L vs. -2.9 ± 4.1 mmol/L at T/HS; p<0.005) and increase in lactate (baseline 1.4 ± 0.7 mmol/L vs. 6.3 ± 1.7 mmol/L at T/HS; p<0.0001). We also observed a significant increase in plasma thrombin-antithrombin (TAT) complex levels (baseline 10.31 ± 12.02 ng/mL vs. T/HS 118.6 ng/mL ± 27.57 ng/mL; p<0.001) and HMGB-1 (baseline 0.76 ± 2.29 ng/mL vs. T/HS 5.76 ± 5.51 ng/mL; p <0.0001) after induction of T/HS (**Figures 2B, C**, respectively). Significant increases in organ injury were identified between sham and T/HS groups after resuscitation with LR as determined by H&E staining and blinded pathology scoring for both lungs (sham 2.7 ± 1.1 vs. T/HS 4.7 ± 2.6; p<0.05) and kidneys (sham 1.3 ± 2.3 vs. T/HS 7.0 ± 2.1; p<0.01) (**Figures 2D, E**, respectively).

### Dekaparin does not worsen development of hemorrhagic shock or prevent LR-resuscitation-mediated reversal of hemorrhagic shock

**Figure 3:**
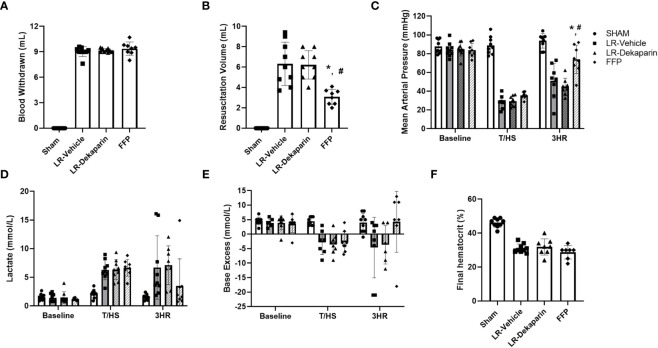
Dekaparin does not alter induction or LR-mediated reversal of hemorrhagic shock. **(A)** The volume of blood withdrawn in mL required to reach a target mean arterial pressure (MAP) of 35 mmHg. **(B)** The volume (mL) of resuscitation fluid required to restore hemodynamic stability. **(C)** MAP was continuously monitored using PowerLab. **(D)** Blood lactate (mmol/L), **(E)** base excess (mmol/L), and **(F)** hematocrit (%) were measured by iSTAT. Data are expressed as mean with standard deviation. N=9 Sham, N=9 LR-vehicle, N=9 LR-dekaparin, N=8 FFP. * denotes p<0.05 compared to LR-vehicle. # denotes p<0.05 compared to LR-dekaparin. LR, Lactated Ringer's; FFP, fresh frozen plasma; T/HS, trauma and hemorrhagic shock; 3HR, 3 hours after resuscitation.

In order to determine if the anticoagulant activity of dekaparin had a detrimental effect on progression of shock, we compared hemodynamics, metabolic indicators of shock, and bleeding and resuscitation volumes between groups. Pretreatment with dekaparin did not affect the estimated percent of total blood volume withdrawn to reach the target MAP of 35 mmHg (LR-vehicle 46.4 ± 4.2% of circulating blood volume vs. LR-dekaparin 46 ± 0.02%, FFP 45.9 ± 0.04% of circulating blood volume; p>0.05), nor did it affect the overall volume of blood withdrawn to achieve hemorrhagic shock (LR-vehicle 9.0 mL ± 0.5 mL, LR-dekaparin 9.2 ± 0.2 mL, FFP 9.4 ± 0.8 mL; p>0.05) (**Figure 3A**). While resuscitation volumes required to reverse shock were lowest among FFP-resuscitated animals, as expected, volumes required for hemodynamic stabilization among LR-resuscitated animals were not affected by the administration of dekaparin. (6.3 ± 2.0 mL vs. 6.2 ± 1.3 ml, respectively; p>0.05) (**Figure 3B**). Furthermore, while resuscitation with FFP resulted in improved recovery of MAP compared to LR resuscitation, no differences were observed between vehicle and dekaparin-treated animals (**Figure 3C**). Similarly, dekaparin had no effects on blood lactate or BE values following induction of T/HS or after resuscitation (**Figures 3D, E**, respectively). Additionally, no differences in final hematocrit were observed between groups (LR-vehicle 31.0 ± 2.6%, LR-dekaparin 31.9 ± 4.8%, FFP 28.7 ± 3.9%; p>0.05) (**Figure 3F**). Finally, no macroscopically evident bleeding was noted during organ collection among animals treated with dekaparin.

### Dekaparin attenuates trauma and HS-associated lung and kidney injury

**Figure 4:**
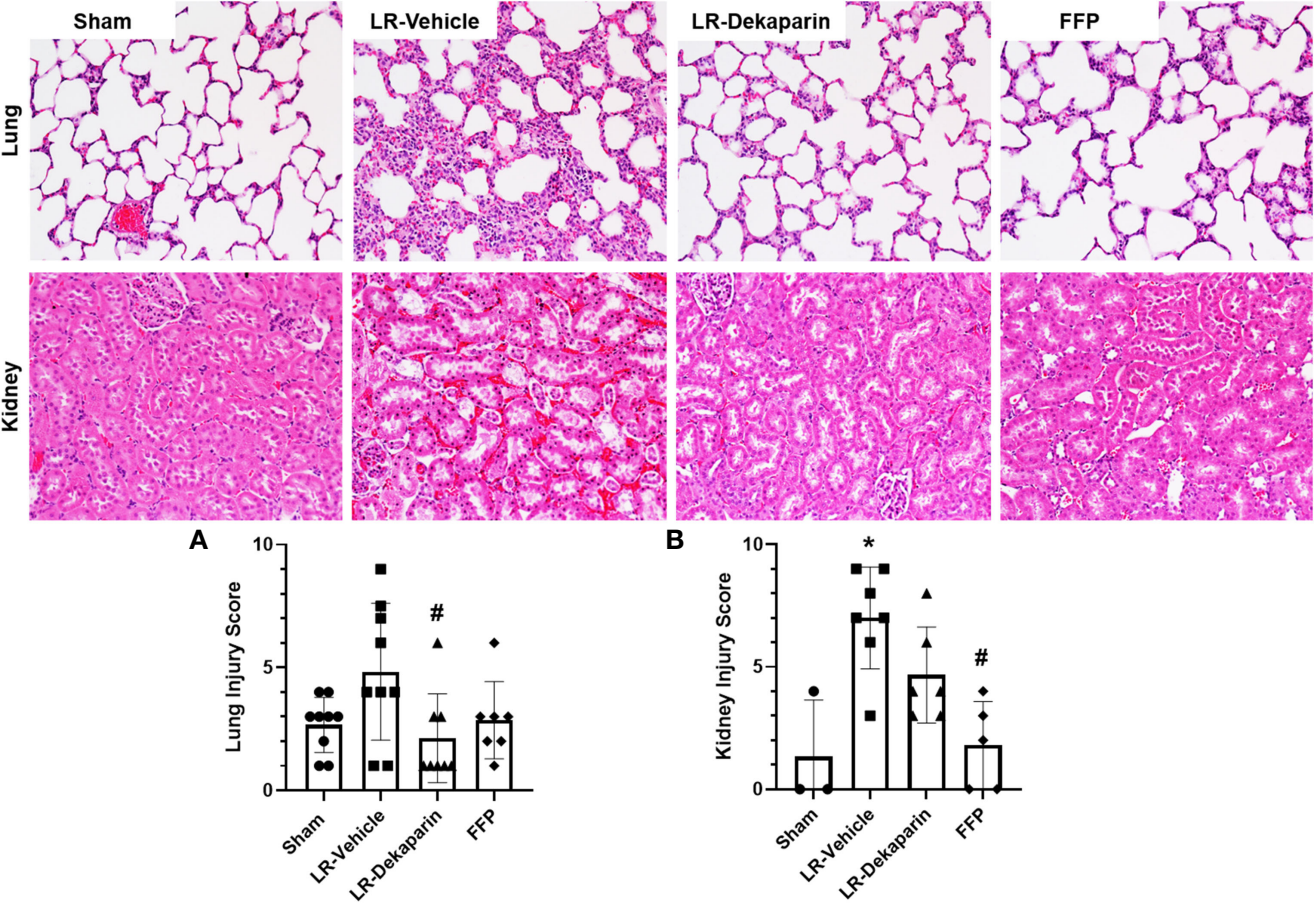
Dekaparin attenuates T/HS-induced lung and kidney injury. Lung and kidney tissue were collected after 3HR for hematoxylin and eosin (H&E) staining and assessment of organ injury by a blinded pathologist. Representative H&E-stained tissue images (20x) are provided for both lung (top) and kidney (bottom). **(A)** Lung and **(B)** kidney injury scores were quantified to compare between groups. Data are expressed as mean with standard deviation. For lungs, N=9 Sham, N=9 LR-vehicle, N=8 LR-dekaparin, N=7 FFP. For kidneys, N=3 Sham, N=7 LR-vehicle, N=6 LR-Dekaparin, N=5 FFP. N=7-9 for all groups. * denotes p<0.05 compared to sham. # denotes p<0.05 compared to LR-vehicle. LR, Lactated Ringer's; FFP, fresh frozen plasma; T/HS, trauma and hemorrhagic shock; 3HR, 3 hours after resuscitation.

Lung and kidney tissues were H&E stained and assessed for the presence of organ injury by a board-certified pathologist blinded to treatment group. Tissues were scored based on a 4-point organ injury scoring system (**Supplemental Tables 1, 2**). Representative tissue images are provided. Pronounced induction of pulmonary congestion and alveolar thickening was demonstrated in the LR-vehicle group compared to sham animals (**Figure 4A**). We observed a significant reduction in lung injury scores after resuscitation with LR among dekaparin-treated animals compared to vehicle. Interestingly, there were no significant differences in the degree of lung injury between LR-dekaparin and FFP groups, which were both similar to sham animals (**Figure 4A**). Similarly, we observed significant increase in kidney injury scores in the LR-vehicle animals compared to sham animals, which was not apparent in the LR-dekaparin group (**Figure 4B**). While the FFP-treated animals exhibited a further reduction in kidney injury scores that were significantly decreased from LR-vehicle, no differences between LR-dekaparin and FFP groups were noted.

### Lung and kidney neutrophil infiltration are reduced in rats receiving dekaparin compared to vehicle

**Figure 5:**
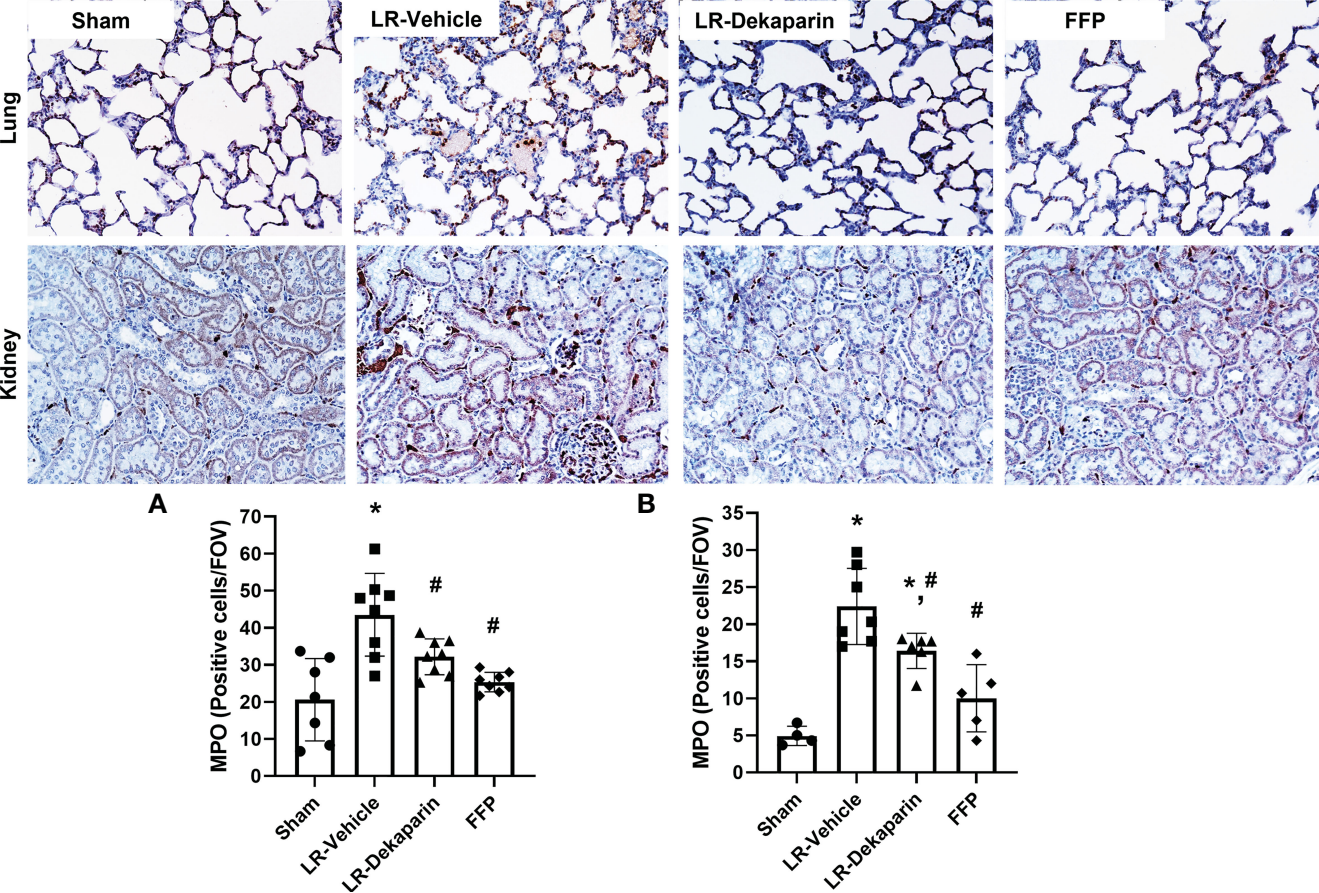
Dekaparin reduces organ neutrophil recruitment after T/HS. Lung and kidney tissue were collected after 3HR for immunohistochemical staining with anti-myeloperoxidase (MPO) antibodies. Representative images (20x) are provided for lung (top) and kidney (bottom). Three random images were collected of the **(A)** lungs and **(B)** cortex region of kidneys for quantifying the number of MPO-positive cells per field of view by a technician blinded to treatment group. Data are expressed as mean with standard deviation. For lungs, N=7 Sham, N=8 LR-vehicle, N=8 LR-dekaparin, N=8 FFP. For kidneys, N=3 Sham, N=7 LR-vehicle, N=6 LR-Dekaparin, N=5 FFP. * denotes p<0.05 compared to sham. # denotes p<0.05 compared to LR-vehicle. LR, Lactated Ringer's; FFP, fresh frozen plasma; T/HS, trauma and hemorrhagic shock; 3HR, 3 hours after resuscitation.

Lung and kidney tissue were stained with anti-MPO antibodies to quantify neutrophil infiltrates in response to T/HS. Representative immunohistochemistry (IHC) images are provided. We found that compared to sham, LR-vehicle-treated animals exhibited significant increases in both lung (**Figure 5A**) and kidney (**Figure 5B**) neutrophil infiltrates, which were significantly reduced in the LR-dekaparin group. While a further reduction in organ neutrophil abundance in the FFP group was appreciated, there was no significant difference between FFP and LR-dekaparin groups.

### Lung microvascular fibrin deposition is reduced in dekaparin-treated rats after T/HS

**Figure 6:**
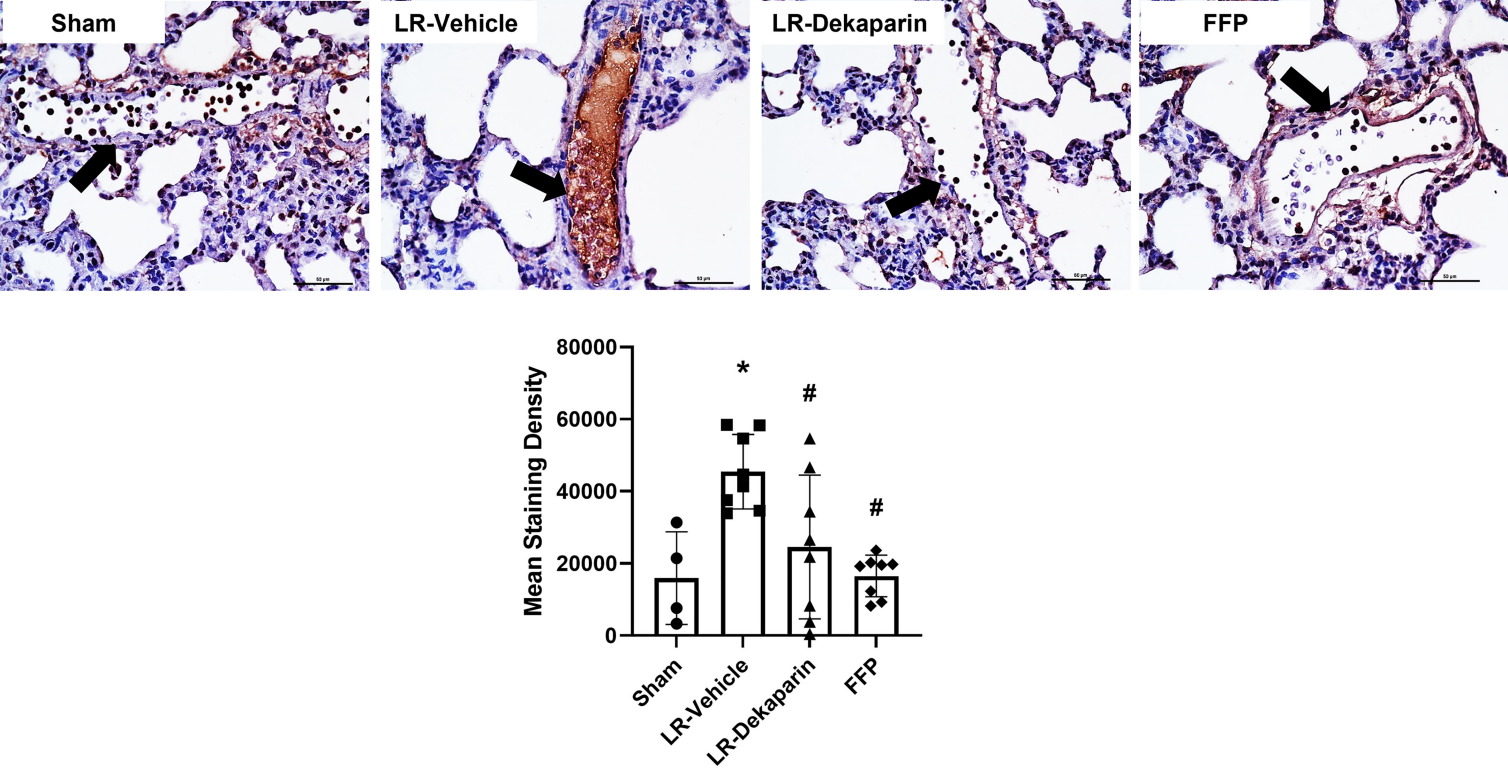
Dekaparin reduces T/HS-associated intravascular fibrin deposition. Lung tissue was collected after 3HR for immunohistochemical staining with anti-fibrin antibodies. Representative images (40x) images are provided. Three images were taken of each tissue and staining density quantified using NIS Elements software by a technician blinded to treatment group. Data are expressed as mean with standard deviation. N=4 Sham, N=8 LR-vehicle, N=8 LR-Dekaparin, N=8 FFP. Arrows indicate blood vessels. Scale bar denoted 50 μm. * denotes p<0.05 compared to sham. # denotes p<0.05 compared to LR-vehicle. LR, Lactated Ringer's; FFP, fresh frozen plasma; T/HS, trauma and hemorrhagic shock; 3HR, 3 hours after resuscitation.

Tissues were stained with anti-fibrin antibodies to quantify fibrin formation and deposition following T/HS. No positive fibrin staining was detected in kidney tissue (data not shown). Representative lung images are provided. Compared to sham, we observed significant increases in intravascular fibrin staining in LR-vehicle animals (**Figure 6**). Lung fibrin staining was significantly decreased in LR-dekaparin-treated animals compared to vehicle. A further reduction in fibrin formation was noted in FFP-resuscitated animals; however, no differences between FFP and LR-dekaparin groups were identified.

### Dekaparin treatment decreases plasma levels of thromboinflammatory mediators after T/HS

**Figure 7:**
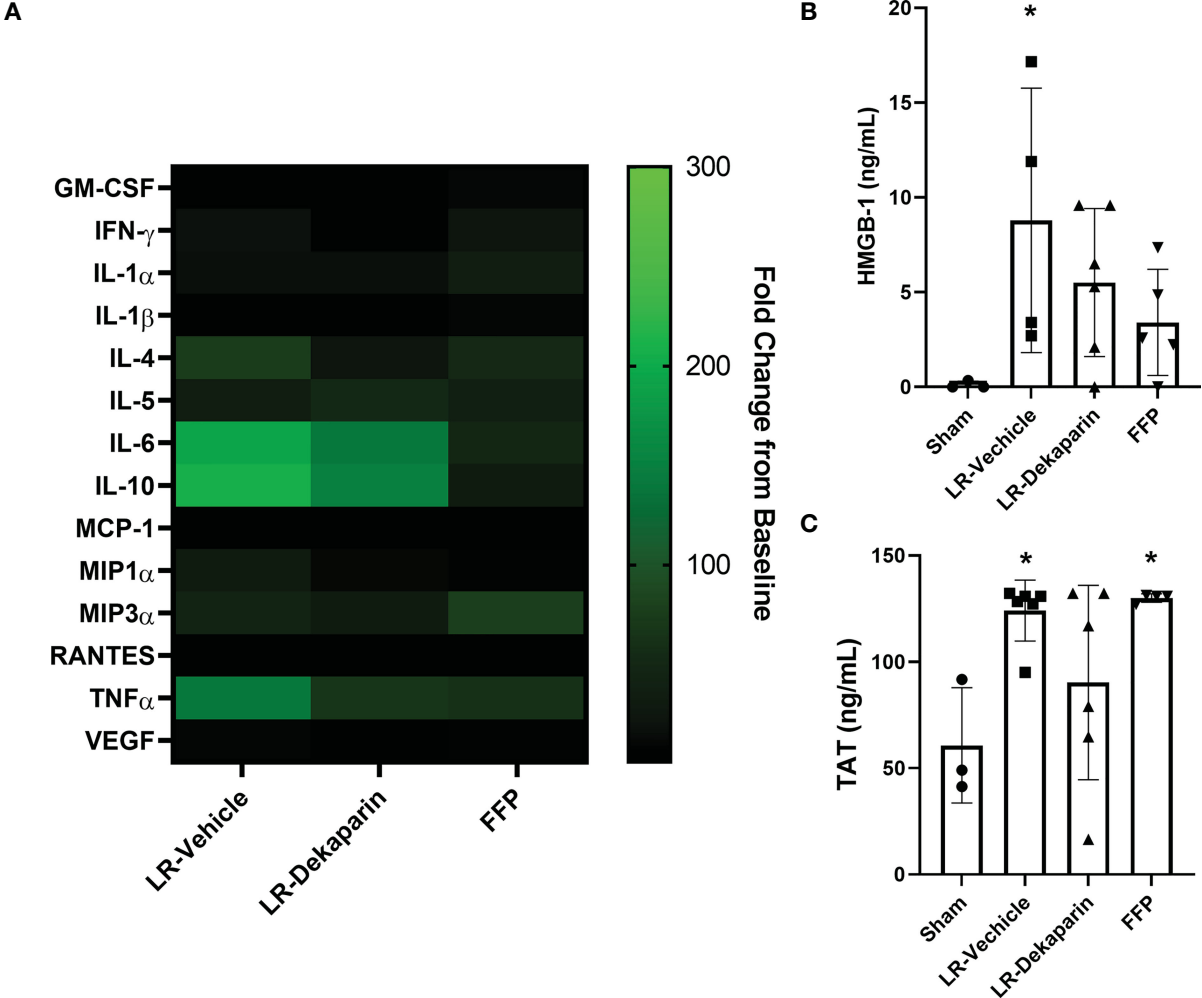
Dekaparin treatment decreases plasma levels of thromboinflammatory mediators after T/HS. **(A)** Cytokine and chemokines were quantified in the baseline and 3HR plasma sample by Bio-Plex Pro Rat Cytokine 23-Plex Assay. Analysis was restricted to N=4 per group due to assay cost. Markers that were undetectable were not included in the analysis. Data at 3HR were normalized to baseline values and expressed fold change over baseline in a heat map. **(B)** High mobility group box-1 (HMGB-1) and **(C)** thrombin-antithrombin (TAT) complex levels were measured in 3HR plasma samples by enzyme-linked immunosorbent assay (ELISA). For HMGB-1 ELISA: N=3 Sham, N=4 LR-vehicle, N=6 LR-Dekaparin, N=5 Sham. For TAT ELISA: N=3 Sham, N=6 LR-vehicle, N=6 LR-Dekaparin, N=4 FFP. Data are expressed as mean with standard deviation. * denotes p<0.05 compared to sham. LR=Lactated Ringer's; FFP=fresh frozen plasma; T/HS, trauma and hemorrhagic shock; 3HR, 3 hours after resuscitation; IL, interleukin; GM-CSF, granulocyte-macrophage colony-stimulating factor; IFN, interferon; MCP-1, monocyte chemoattractant protein-1; MIP, macrophage inflammatory protein; RANTES, regulated upon activation, normal T cell expressed and secreted; TNF, tumor necrosis factor; VEGF, vascular endothelial growth factor.

To determine whether dekaparin treatment modulates the thromboinflammatory milieu after induction of tissue trauma, hemorrhagic shock, and resuscitation, we used 3HR samples to examine plasma levels of cytokines, chemokines, damage-associated molecular patterns, and TAT complex. While no statistically significant differences were identified between groups, notable trends demonstrated a qualitative reduction in several cytokines and chemokines when comparing vehicle to dekaparin-treated animals (**Figure 7A**, heat map). As expected, values were lowest among animals resuscitated with FFP. Further, we found that while the LR-vehicle group demonstrated significant increases in HMGB-1 after T/HS, there was no significant difference in LR-dekaparin or FFP groups compared to sham (**Figure 7B**). Finally, while we observed significant increases in TAT complex levels after induction of T/HS and resuscitation in the LR-vehicle and FFP groups, no significant increases in TAT levels among LR-dekaparin animals were noted (**Figure 7C**).

## Discussion

Injury to the vital organs, most commonly the lungs and kidneys, and subsequent MODS is common after T/HS and associated with rapid recruitment of neutrophils to post-ischemic tissues (1, 35). Systemic activation of the coagulation system is a hallmark of T/HS that can both augment inflammation and, in turn, be further amplified by inflammation in a cyclical process known as thromboinflammation (5, 35, 36). However, safely targeting thromboinflammation in the critically ill is a therapeutic challenge. Recent work demonstrates that dekaparin can attenuate liver injury in a rodent model of ischemia/reperfusion-induced organ damage (7, 13). The goal of our study was to determine whether dekaparin treatment could attenuate thromboinflammation and multiple organ injury secondary to T/HS. Vehicle or dekaparin was administered prior to induction of T/HS and resuscitation with LR. Treatment groups were compared to animals resuscitated with FFP, as FFP has known therapeutic effects for mitigating T/HS-induced organ injury in animal models (22, 37, 38). We found that compared to vehicle, animals treated with dekaparin demonstrated a marked reduction in both lung and kidney tissue injury, which was associated with a decrease in both local and systemic markers of coagulation and inflammation. Findings in LR-dekaparin animals were remarkably similar to those achieved with FFP resuscitation, indicating dekaparin has similar anti-thromboinflammatory and organ protective benefits to FFP. In spite of its anticoagulant activity, dekaparin did not negatively impact hemodynamic stability during or recovery from HS and was not associated with intestinal or other organ macroscopic bleeding.

HSPGs play a vital role in vascular barrier function, immune cell adhesion/tethering, hemostasis, and overall maintenance of cardiovascular homeostasis (6). In particular, 3-OS-HSPG plays an important role in regulating the anticoagulant and anti-inflammatory activity of the endothelium through its interaction with AT. While only a fraction of HSPGs are endowed with the 3-OS modification, these findings add to the recent literature demonstrating the important biological role and therapeutic potential of 3-OS-HSPG and dekaparin, respectively. As endothelial production of 3-OS-HSPG is controlled by the *HS3ST1* gene, Smits et al. used *HS3ST1^-/-^* mice to demonstrate that loss of 3-OS-HSPG results in accelerated lethality and enhanced sensitivity to inflammatory insult in septic shock models (9). More recently, Arnold et al. showed that administration of dekaparin prior to sterile inflammatory injury *via* ischemia-reperfusion significantly attenuated liver damage (7, 13). This was associated with abrogation of systemic coagulation and a reduction in liver neutrophil infiltration, similar to results observed in our study. Further, Arnold and colleagues demonstrated that dekaparin and similar 3-*O*-sulfated heparan sulfate oligosaccharides can bind to and sequester circulating HMGB-1, a known mediator of organ injury during sterile inflammation (7, 13). In agreement with this, we found that while T/HS resulted in significant induction of plasma HMGB-1 in the LR-vehicle group, no such increase was observed in the LR-dekaparin group. Interestingly, we observed that dekaparin treatment prevented T/HS-induced induction of TAT complex, a protective effect that was not apparent with FFP resuscitation. This would seem in disagreement with our past findings demonstrating that FFP resuscitation attenuates T/HS-induced hypercoagulability; however, our previous study assessed enzymatic thrombin generation by calibrated automated thromogram, which measures thrombin generating potential, unlike TAT complex, which measures thrombin that has already been generated (23). We elected to measure TAT complex in this study given that dekaparin was administered as a pretreatment, which we observed resulted in attenuated production of TAT complexes. Our findings are likely explained by the fact that thrombin generation is upregulated rapidly after T/HS and TAT complexes are still circulating even after resuscitation with FFP.

Our data are also in agreement with past findings that highlight a role for restoring, or perhaps augmenting, the biological function of essential HSPGs as a viable therapeutic avenue for mitigating organ injury in the critically ill. HSPGs are prominently shed from the endothelial surface in response to systemic inflammation or injury, and circulating HSPGs, such as syndecan-1, are known predictive biomarkers for poor outcomes after T/HS (15). There are extensive data in the literature from our group and others that indicate early restoration of endothelial HSPGs through strategies such as FFP resuscitation can improve organ function (22, 37–39). While these experiments did not determine whether dekaparin targets or homes to the endothelium, they reinforce the important biological role of 3-OS-HSPG during critical illness and the potential for targeted strategies that can increase endothelial expression of 3-OS-HSPG (8, 40). Our future investigations seek to quantify changes in EC expression of 3-OS-HSPG after T/HS, modulation of enzymes that control production of 3-OS-HSPG, and whether molecules like dekaparin can be used to restore normal EC function.

Finally, these studies provide strong premise to support the future development of synthetic heparan sulfates, such as dekaparin, for therapeutic use. Notably, heparin and low-molecular-weight heparin (LWMH) are highly sulfated heparan sulfate analogs that are commonly administered for in-hospital thromboprophylaxis in the critically ill. However, pharmaceutical heparins are animal-derived and, therefore, susceptible to contamination issues, with devastating past consequences, and have widely varying molecular structures (41). This is likely a key reason why although several studies have noted that heparin administration has modest coagulation-dependent immunomodulatory effects and may confer a survival benefit during severe sepsis, results are highly variable and inconclusive at best (42, 43). In addition, therapeutic doses of anticoagulants like LMWH are contraindicated in this population due to a high risk of bleeding. Synthetic heparan sulfates allow for tailoring of oligosaccharides that have specific chain lengths and sulfate modifications to achieve unique, consistent therapeutic effects. For instance, past experiments utilizing dekaparin, and other synthetic heparan sulfate molecules, have conclusively demonstrated that heparan sulfate molecules with both anticoagulant *and* anti-inflammatory activity are essential for organ protection in animal models. Molecules with only anticoagulant or anti-inflammatory properties had minimal therapeutic benefit (13). Furthermore, dekaparin has similar anticoagulant activity and mechanisms of action to fondaparinux; however, unlike fondaparinux, dekaparin can safely be reversed using protamine (17). Thus, synthetic molecules, like dekaparin, could provide a future alternative strategy for thromboprophylaxis in recovering trauma patients with the added benefit of anti-inflammatory properties. Lastly, as 3-OS-HSPG plays an essential role in regulating infectivity of pathogens like herpes simplex virus, development of synthetic dekaparin could have broad implications toward improved understanding of viral pathogenesis and developing therapeutics to sequester circulating blood-borne virions (44).

### Limitations

There are several limitations to this study. First, treatments were administered prior to induction of T/HS and, therefore, only assessed protective effects of dekaparin. Future studies will need to be performed to determine if dekaparin can reverse T/HS-induced organ injury. Second, our observations on the therapeutic potential of dekaparin are restricted to rodent models that do not perfectly recapitulate human MODS after T/HS. In addition, our analysis was restricted to the acute phase post-T/HS in rodents maintained under anesthesia with temperature regulation, and results could differ in long-term studies in animals that have regained consciousness and are self-regulating body temperature. Also, our analyses only included male rats, and, given known sex dimorphisms in response to T/HS, further studies will utilize males and females. While microvascular thrombosis is a known mediator of organ injury in the critically ill (45), no organized thrombi were noted on lung or kidney histopathology. Additionally, gross organ assessments were performed to examine any effect of dekaparin on bleeding; however, microscopic examinations in the future will further define this potential adverse effect of dekaparin. Thus, fibrin deposition was measured by immunostaining as a surrogate for localized coagulation in the organs. Further, our studies did not address whether dekaparin can modulate endothelial anticoagulant or anti-inflammatory signaling or augment endothelial localization of AT. This will be the subject of future *in vitro* studies. In addition, while it is unclear from our data whether the therapeutic anticoagulant or anti-inflammatory properties of dekaparin predominate in the setting of T/HS-induced organ injury, addressing this will be the subject of future studies. Finally, this study employed a model of controlled hemorrhage, and dekaparin was administered to hemodynamically stable animals. Future studies will examine the effects of dekaparin for preventing or reversing organ injury in models of uncontrolled hemorrhage for which dekaparin could modulate hemostasis.

## Conclusions

In conclusion, our results show that administration of a 3-OS-heparan sulfate mimetic can mitigate early T/HS-induced organ injury *via* attenuation of localized and systemic thromboinflammation. These findings demonstrate the important role of 3-OS-HSPGs for modulating thromboinflammation following T/HS and justify the future development of synthetic anticoagulant and anti-inflammatory heparan sulfates for potential clinical use.

## Data availability statement

The raw data supporting the conclusions of this article will be made available by the authors, without undue reservation.

## Ethics statement

The animal study was reviewed and approved by Institutional Animal Care and Use Committee of The University of Texas Health Science Center at Houston.

## Author contributions

MV designed the research, performed experiments, analyzed data, and wrote the manuscript. BO performed experiments and edited the manuscript. KL performed experiments and edited the manuscript. MM performed experiments and edited the manuscript. Y-WW performed experiments and edited the manuscript. VP performed experiments and edited the manuscript. JR analyzed data, interpreted data, and edited the manuscript. YX synthesized and characterized dekaparin. KA assisted with study design, performed experiments, and wrote the manuscript. JL assisted with study design and edited the manuscript. JC conceived the project, designed the research, directed the project, analyzed data, interpreted data, and wrote the manuscript. All authors contributed to the article and approved the submitted version.
