# Supplementary material for: A 3-O-sulfated heparan sulfate dodecasaccharide (12-mer) suppresses thromboinflammation and attenuates early organ injury following trauma and hemorrhagic shock
Source: Front Immunol. 2023 Apr 14;14:1158457. doi: 10.3389/fimmu.2023.1158457 (PMC10140401; doi:10.3389/fimmu.2023.1158457)
Supplement: Supplementary file 1 [file DataSheet_1.docx]

Supplementary Material

**Supplemental Table 1.** Lung injury scoring ranging in severity from 0 (no injury) to 3 (severe).

| **Lung Injury** | **Score Description** | | | |
| --- | --- | --- | --- | --- |
|  | **0** | **1** | **2** | **3** |
| **Peribronchial inflammatory cell infiltration** | None | Prominent germinal centers of lymphoid follicles | Infiltration between lymphoid follicles | Confluent band-like form |
| **Alveolar septal infiltration** | None | Minimal | Moderate | Severe, impeding of lumen |
| **Thickness** | None | Mild thickening | Clearly thickened walls | Thickening of the wall, with 50–100% extremely thick |
| **Alveolar edema** | None | Focal | In multiple alveoli | Widespread, involving lobules |
| **Alveolar exudate** | None | Focal | In multiple alveoli | Prominent, widespread |
| **Percentage of affected lung tissue** | 0% | 5% to 25% | 26% to 50% | >50% |

**Supplemental Table 2.** Kidney injury scoring ranging in severity from 0 (no injury) to 3 (severe).

| **Kidney Injury** | **Score Description** | | | |
| --- | --- | --- | --- | --- |
|  | **0** | **1** | **2** | **3** |
| **Interstitial inflammatory cell infiltration** | None | Focal | Aggregates | Diffuse |
| **Tubular simplification** | None | Minimal | Moderate | Severe |
| **Desquamation** | None | Minimal | Moderate | Severe |
| **Percentage of affected tissue** | 0% | 5% to 25% | 26% to 50% | >50% |
